# FOXM1D potentiates PKM2‐mediated tumor glycolysis and angiogenesis

**DOI:** 10.1002/1878-0261.12879

**Published:** 2021-04-02

**Authors:** Wei Zhang, Xin Zhang, Sheng Huang, Jianfeng Chen, Peipei Ding, Qi Wang, Luying Li, Xinyue Lv, Ling Li, Pingzhao Zhang, Danlei Zhou, Wenyu Wen, Yiping Wang, Qun‐Ying Lei, Jiong Wu, Weiguo Hu

**Affiliations:** ^1^ Fudan University Shanghai Cancer Center and Institutes of Biomedical Sciences Shanghai Medical College Fudan University Shanghai China; ^2^ Department of Breast Surgery Breast Cancer Institute Fudan University Shanghai Cancer Center Shanghai Medical College Fudan University Shanghai China; ^3^ Key Laboratory of Breast Cancer in Shanghai Fudan University Shanghai Cancer Center Fudan University Shanghai China

**Keywords:** angiogenesis, exosome, FOXM1D, glycolysis, NF‐κB, PKM2

## Abstract

Tumor growth, especially in the late stage, requires adequate nutrients and rich vasculature, in which PKM2 plays a convergent role. It has been reported that PKM2, together with FOXM1D, is upregulated in late‐stage colorectal cancer and associated with metastasis; however, their underlying mechanism for promoting tumor progression remains elusive. Herein, we revealed that FOXM1D potentiates PKM2‐mediated glycolysis and angiogenesis through multiple protein–protein interactions. In the presence of FBP, FOXM1D binds to tetrameric PKM2 and assembles a heterooctamer, restraining PKM2 metabolic activity by about a half and thereby promoting aerobic glycolysis. Furthermore, FOXM1D interacts with PKM2 and NF‐κB and induces their nuclear translocation with the assistance of the nuclear transporter importin 4. Once in the nucleus, PKM2 and NF‐κB complexes subsequently augment *VEGFA* transcription. The increased VEGFA is secreted extracellularly via exosomes, an event potentiated by the interaction of FOXM1 with VPS11, eventually promoting tumor angiogenesis. Based on these findings, our study provides another insight into the role of PKM2 in the regulation of glycolysis and angiogenesis.

## Graphical abstract

FOXM1D binds to tetrameric PKM2 and assembles a heterooctamer, thereby promoting aerobic glycolysis. Further, FOXM1D interacts with PKM2 and NF‐κB and induces their nuclear translocation via importin 4. The nuclear PKM2 and NF‐κB complexes subsequently augment *VEGFA* transcription. The increased VEGFA is secreted extracellularly via exosomes, an event potentiated by the interaction of FOXM1 with VPS11, eventually promoting tumor angiogenesis.

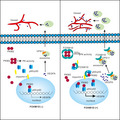

**Abbreviations**
ADP: adenosine diphosphate
ATP: adenosine triphosphate
ECAR: extracellular acidification
EGF: epidermal growth factor
EMT: epithelial–mesenchymal transition
FBP: fructose‐1,6‐biphosphate
FOXM1: forkhead box M1
HIF‐1α: hypoxia‐inducible factor 1
HUVEC: human umbilical vein endothelial cell
NF‐κB: nuclear factor kappa‐light‐chain‐enhancer of activated B cells
OCR: oxygen consumption rate
PEP: phosphoenolpyruvate
PK: pyruvate kinase
PKM2: pyruvate kinase M2
PTM: post‐translation modification
ROCK1: Rho‐associated protein kinase 1
VEGFA: vascular endothelial growth factor‐A

## 1 Introduction

Late‐stage cancer frequently indicates a large tumor size accompanied by metastasis to nearby tissues, lymph nodes, or even other distant organs. This then leads to a poor therapeutic response and prognosis due to the limited number of available treatment regimens. Therefore, the investigation of the late‐stage cancer still presents an unmet need. Angiogenesis, the process of forming new vascular networks, is required to supply adequate oxygen and nutrients to support tumor proliferation and further metastasis [1]. The production of nutrients and the formation of vasculature are prerequisites for the uncontrolled and rapid proliferation that is a hallmark of cancer cells. In order to sustain this level of metabolism, cancer cells must convert large amounts of glucose to lactate even in the presence of oxygen, a process that is termed the Warburg effect or aerobic glycolysis [2, 3]. This may facilitate tumor growth by producing more glucose metabolites for the synthesis of macromolecules [2, 4, 5, 6]. Pyruvate kinase (PK) regulates the final step of glycolysis by transferring a phosphate group from phosphoenolpyruvate (PEP) to adenosine diphosphate (ADP) to produce pyruvate and adenosine triphosphate (ATP) [7]. Due to alterative splicing [8, 9], there are four PK isoforms [10, 11], that is, PKM1, PKM2, PKL, and PKR, in which PKM2 is the primary type expressed and upregulated in tumor cells [12]. In the cytoplasm, PKM2 normally presents in either a homotetramer form that allows for the highly effective conversion of glucose to lactate, acting as a metabolite kinase, or a low activity homodimer form that uses PEP as a phosphate donor to phosphorylate tyrosine residues, acting as a signal transducer and activator of transcription [10, 13]. Upon upstream stimulus, such as epidermal growth factor (EGF) or interleukin‐3, PKM2 also translocates into the nucleus to play a nonmetabolic role as a histone kinase and/or a transcriptional coactivator, thus promoting the Warburg effect and cell cycle progression via cyclin D1 and β‐catenin [14, 15, 16, 17, 18].

In order to facilitate angiogenesis, cancer cells frequently overexpress pro‐angiogenic factors, for example, vascular endothelial growth factor‐A (VEGFA), thereby positioning anti‐angiogenic agents as potential strategies for tumor therapy. However, the current drugs targeting the VEGF pathway, including bevacizumab and sorafenib, have displayed only a limited antitumor effect in certain settings [19], prompting the need to understand the contribution of angiogenesis in tumors and the molecular mechanisms by which it acts. Hypoxia‐inducible factor 1 (HIF‐1α) has shown indication as one of the important links between angiogenesis and glycolysis. HIF‐1α promotes PKM2 transcription, and inversely, PKM2 interaction with HIF‐1α enhances HIF‐1α‐dependent gene transcription [20, 21]. Furthermore, in hypoxic pancreatic tumors both HIF‐1α and NF‐κB together regulate the transcription and secretion of VEGFA, which is controlled by PKM2 translocated to the nucleus [22]. In addition, it has been reported that PKM2 regulates angiogenesis mediated by the NF‐κB p65 subunit, although the underling mechanisms remain largely obscured [23].

Forkhead box M1 (FOXM1) was initially defined as a transcription factor by containing a common DNA‐binding domain termed the forkhead box [24]. It is strongly upregulating in almost all types of tumors and highly correlating with tumor progression [25, 26]. Due to alternative splicing, there are four FOXM1 isoforms, FOXM1A/B/C/D [27, 28]. FOXM1B/C mainly locate in the nucleus and function as a transcription factor by controlling over 200 genes that are mainly involved in cell proliferation [29, 30]. It has been recognized as a major predictor (actually indiscriminately referred to as FOXM1) of adverse outcomes in cancer by gene landscape analysis [25, 31]. In contrast, FOXM1A/D exist predominantly in the cytoplasm without direct transcription‐regulating functions. FOXM1A has been almost ignored, likely due to its low level of expression [28, 32]. Meanwhile, FOXM1D, which we recently identified is highly expressed only in late‐stage colorectal cancer, was found to promote the cancer epithelial–mesenchymal transition (EMT) and metastasis by interacting with ROCKs [28]. In this study, we found that FOXM1D also interplays with multiple proteins including PKM2, importin 4, NF‐κB, and VPS11, it promoting aerobic glycolysis through assembly a heterooctamer with PKM2, which enhances angiogenesis by increasing the expression and release of VEGF.

## 2 Experimental procedures

### 2.1 Cell culture and reagents

All cell lines were purchased from the Type Culture Collection Cell Bank, Chinese Academy of Sciences. HEK 293T cells were grown in DMEM supplemented with 10% fetal bovine serum and 1% penicillin/streptomycin. HeLa cells were grown in 1640 medium supplemented with 10% fetal bovine serum and 1% penicillin/streptomycin. SW‐480 cells were grown in L‐15 medium supplemented with 10% fetal bovine serum and 1% penicillin/streptomycin. LoVo cells were grown in F‐12K Medium supplemented with 10% fetal bovine serum and 1% penicillin/streptomycin. Human umbilical vein endothelial cells (HUVEC) were grown in F‐12K Medium supplemented with 10% fetal bovine serum and 1% penicillin/streptomycin. All cells were cultured at 37 °C with 5% CO_2_. PKM2 was purchased from Abcam (ab89364, Cambridge, MA, USA). All antibodies used in this study are listed in Table S1.

### 2.2 Cell transfection

HeLa or 293FT Cells were planted in a 10‐cm dish for 50% density the day before transfection. Transfection could be performed when the cells have grown to a density of 70%. Plasmid or siRNA was mixed with Lipofectamine 3000 (Invitrogen, Carlsbad, CA, USA) based on the manufacture's guide, incubated for 10 min at room temperature, and then added them into the cell culture dish. Cells were collected 48 h after transfection.

### 2.3 Western blot analysis

Cells were harvested by scraping into an SDS sample buffer containing a cocktail of protease inhibitors and PhosSTOP Phosphatase Inhibitor (Roche, Pleasanton, CA, USA). Western blotting was conducted according to the standard procedure.

### 2.4 Quantitative real‐time PCR

Total RNA from cells was extracted using TRIzol reagent (Invitrogen, Grand Island, NY, USA) and then transcribed into cDNA using a Reverse Transcription System (Promega, Madison, WI, USA). The input cDNA was standardized and amplified for 40 cycles with SYBR Green Master Mix (Invitrogen) and gene‐specific primers on a Roche Light Cycler 480 system (Roche, Basel, Switzerland). We used the *ACTB* gene encoding β‐actin as the endogenous control, and the samples were analyzed in triplicate. All primers used in this study are listed in Table S2.

### 2.5 Extracellular acidification and oxygen consumption rate assays

The extracellular acidification (ECAR) rate and cellular oxygen consumption rate (OCR) were determined using the Seahorse XFe 96 Extracellular Flux Analyzer (Seahorse Bioscience, North Billerica, MA, USA). Experiments were performed according to the manufacturer's protocols. ECAR and OCR were examined using the Seahorse XF Glycolysis Stress Test Kit (Agilent Technologies, Wilmington, DE, USA) and the Seahorse XF Cell Mito Stress Test Kit (Agilent Technologies), respectively. Briefly, 1 × 10^4^ cells per well were seeded into a Seahorse XF 96 cell culture microplate. Baseline measurements were taken, after which glucose, the oxidative phosphorylation inhibitor oligomycin, and the glycolytic inhibitor 2‐DG were sequentially injected into each well at indicated time points for the measurement of ECAR. Oligomycin, the reversible inhibitor of oxidative phosphorylation FCCP (*p*‐trifluoromethoxy carbonyl cyanide phenylhydrazone), and the mitochondrial complex I inhibitor rotenone, plus the mitochondrial complex III inhibitor antimycin A (Rote/AA), were sequentially injected for the measurement of OCR. Data were assessed using the seahorse xf‐96 wave (Agilent Technologies) software. The OCR is shown in pmols·min^−1^ and ECAR in mpH·min^−1^.

### 2.6 Co‐immunoprecipitation assays

HeLa and HEK293T cells were seeded in 10‐cm dishes, and HEK293T cells were cotransfected with the indicated plasmids. Two days following the transfection, the cells were lysed and co‐immunoprecipitated using the antibody or label‐tagged affinity gel, according to the manufacturer's instructions. The immunoprecipitations were washed four times with lysis buffer and stored at −80 °C until needed, or were directly boiled with sigma sample buffer and subsequent SDS/PAGE and sliver staining or western blotting, as described above.

### 2.7 Immunofluorescence (IF) staining and confocal microscopy

HeLa cells grown on the culture slides (BD Biosciences, San Jose, CA, USA) were maintained in a 24‐well cell culture cluster and transfected using Lipofectamine 3000. After incubation for 48 h, the cells were fixed with 4% paraformaldehyde and permeabilized with 0.1% Triton X‐100 in PBS for 5–8 min at room temperature followed by incubation in blocking buffer supplemented with 1% BSA for 1 h at room temperature. The cells were then incubated with FLAG mouse monoclonal antibody (1 : 200 dilution) and PKM2/VPS11 rabbit monoclonal antibody (1 : 200 dilution) at 4 °C overnight. The next day, the cells were washed three times in PBS and then stained with Alexa Fluor 488 Goat Anti‐Mouse IgG and Alexa Fluor 594 Goat Anti‐Rabbit IgG (1 : 5000 dilution; Invitrogen) for 2 h at room temperature. After incubation with DAPI to stain the nuclei, the cells were visualized with a confocal laser scanning microscope (Olympus Corporation, Tokyo, Japan).

### 2.8 Glutathione *S*‐transferase (GST) pull‐down and in vitro binding assays

Bacterial‐expressed GST–FOXM1D or control GST bound to MagneGST glutathione particles was incubated with PKM2‐transfected HEK293T cell lysates for 1 h at 4 °C using the MagneGST Pull‐Down System (Millipore, Darmstadt, Germany), according to the manufacturer's instructions. The washed complexes were eluted by boiling in SDS sample buffer before separation by SDS/PAGE, after which the interactions were analyzed by western blotting.

### 2.9 Protein cross‐linking

Cells from 10‐mm dishes were digested and lysed in 500 μL of lysis buffer with protease inhibitor cocktail at 4 °C for 40 min. The lysates were centrifuged at 12 000 ***g*** for 10 min. The supernatants were drawn into two tubes (200 μL each) (one for cross‐link and one for control). A preparation of 0.5% glutaraldehyde solution (Solarbio, Shanghai, China; #G8090) (10 μL) was added to the lysate to a final concentration of 0.0025 wt %. The solution was mixed well and incubated on ice for 5 min. Then, 10 μL of 1 m glycine was added for 15 min at room temperature. Finally, 50 μL of 5 × loading buffer was added to the tube and the lysates were boiled at 100 °C.

### 2.10 Gel chromatography

Cells were seeded in a 10‐cm dish. When a concentration of 80–90% was achieved, the cells were harvested and washed three times with ice‐cold PBS. The lysis buffer contained a protease inhibitor and a phosphatase inhibitor cocktail and lysates were incubated for 1 h before centrifugation at 12 000 ***g*** for 15 min to remove cell debris. The gel chromatography column (Superdex 200; Amersham Biosciences, Marlborough, MA, USA) was washed and equilibrated with cold PBS (4 °C) before passing the extracts over the gel chromatography column. The flow rate was 0.3 mL·min^−1^. Fractions were collected at 0.3 mL per tube and analyzed by western blot. The molecular mass was determined by a Gel Chromatography Calibration Kit HMW (GE Healthcare, Marlborough, MA, USA). For each protein subjected to the polymer formation experiment, 60 μg was used per test and was co‐incubated overnight prior to the gel chromatography assay.

### 2.11 Protein expression and purification

The 293FT cells were transfected with Flag‐tagged FOXM1A, FOXM1D, or FBP plasmid. Two days later, the Flag‐tagged proteins were purified from the cell lysates using Flag‐tagged beads (MedChemExpress, Monmouth Junction, NJ, USA), according to the manufacturer's instructions. Briefly, 10 mg·mL^−1^ Flag peptide was used for competitive binding, in which the volume of the peptide buffer was at least 5‐fold larger than the volume of beads. To achieve highly pure proteins, the eluted proteins were further purified by gel chromatography.

### 2.12 Tumor tissue microangiography

Microangiography for blood vessels was performed using beamline BL13W1, an X‐ray imaging, and biomedical application station at the Shanghai Synchrotron Radiation Facility (SSRF) in China. The maximum light size of the beam was 45 mm (horizontal) by 5 mm (vertical) at the object position at 20 keV. All animals were anesthetized by intraperitoneal injection of ketamine (200 mg·kg^−1^) (Ketanest; Pfizer, Karlsruhe, Germany). The image contrast agent barium sulfate was suspended in glycerol (50% water solution; a concentration of 0.5 g·mL^−1^) and injected into the left ventricle of nude mice. Serial images of tumor blood vessels were then recorded at the SSRF.

### 2.13 Chromatin immunoprecipitation

Chromatin was immunoprecipitated according to the EZ‐Magna G Chromatin Immunoprecipitation kit's (Millipore) user guide. Occupancy was assessed by quantitative PCR for samples precipitated with a specific antibody versus samples precipitated with the control immunoglobulin G (12–371; Millipore) using the specific primers indicated in the related tables.

### 2.14 Human umbilical vein endothelial cell tube formation assay

HeLa cells with ectopic FOXM1A/B/C/D expression were cultured in complete medium for 12 h and then serum‐free medium for 24 h, after which point the supernatants were collected. Matrigel (BD Biosciences, Franklin Lakes, NJ, USA) (50 μL) was pipetted into each well of a 96‐well plate and polymerized for 30 min at 37 °C in incubator. HUVECs (4 × 10^4^ cells) were suspended in 100 μL of the above supernatants and distributed into each well. After incubation for 5 h at 37 °C, images were taken with a 100× and 400× phase‐contrast microscope. Each condition was assessed at least in triplicate in three independent experiments.

### 2.15 Detection of VEGFA in the supernatant and total cell lysate

The cells were cultured in a 6‐well plate at a concentration of 1 × 10^6^ cells per well for 24 h, after which the supernatant was harvested to measure VEGFA concentration using a commercially available fluorescence‐based assay kit (ARIGO, ARG81305, Taiwan, China). In addition, the cells were collected for VEGFA detection by western blot. To explore the function of Golgi in FOXM1D‐mediated exosome release, the cells were treated with 10 μm monensin (Selleck, S2324, Houston, TX, USA) for 3 h in the indicated experiment (Fig. 7D).

### 2.16 Measurement of pyruvate kinase activity

The PK activity was measured using a PK activity assay kit (Solarbio, BC0545) based on the manufacture's guide.

### 2.17 Exosome collection

Cells were plated in a 10‐cm dish with complete medium. When the cell density reached above 95%, the culture medium was changed to serum‐free and collected after 12‐h culture. Exosomes were collected by filtration of the supernatant with a 0.22‐μm membrane and 1 × 10^6^ ***g*** supernatant centrifugation for 2 h.

## 3 Result

### 3.1 Identification of the FOXM1D and PKM2 interaction

**Fig. 1:**
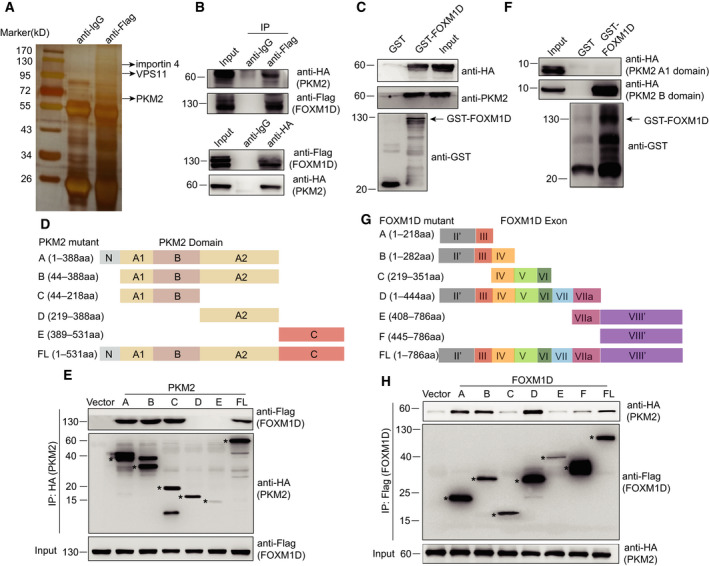
The physical interaction between FOXM1D and PKM2. (A) Identification of FOXM1D‐binding proteins, including PKM2, NF‐κB subunit p65, VPS11, and importin 4 by Co‐IP using rabbit anti‐Flag antibody or the rabbit control IgG isotype antibody, and further by LC‐MS/MS analysis. See also Data S1. (B) Verification of the interaction between Flag‐FOXM1D and HA‐PKM2 by Co‐IP. Top, anti‐Flag antibody captured HA‐PKM2; Bottom, anti‐HA antibody captured Flag‐FOXM1D. (C) GST‐FOXM1D binds to HA‐PKM2 as determined by GST pull‐down assay and immunoblotting assay using anti‐HA and anti‐PKM2 antibodies. (D) Schematic diagram of the truncated mutants of PKM2 based on their structural domain. (E) PKM2 mutants A, B, and C bound to FOXM1D as revealed by Co‐IP assay. (F) GST‐FOXM1D binds to PKM2 B, but not the A1 domain, which were tagged with HA and separately expressed into 293T cells, as determined by GST pull‐down assay. (G) Schematic diagram of the truncated mutants of FOXM1D based on exons. The related constructs only contained the coding fragments in exons II and VIII; therefore, the single quotation marks were added. (H) FOXM1D exons II and III encoded region binds to PKM2 as revealed by Co‐IP assay. Flag‐FOXM1D mutants A, B, and D that bound to HA‐PKM2 share the common exons II and III. FL, full length.

In late‐stage colorectal cancer, FOXM1D, a new isoform of FOXM1, promotes tumor metastasis by binding to and further activating ROCKs [28]. In our study, we found that anti‐Flag antibody immunoprecipitated more proteins, including PKM2 rather than ROCK2, in the co‐immunoprecipitation (Co‐IP) assay and subsequent mass spectrometric analysis of HeLa cells overexpressing Flag‐FOXM1D (Fig. 1A, and Supplemental Data). Next, we confirmed the interaction between PKM2 and FOXM1D by mutual Co‐IP analysis using anti‐Flag and anti‐HA antibodies in 293T cells transfected with HA‐PKM2 and Flag‐FOXM1D (Fig. 1B). The result of GST pull‐down assay also demonstrated this interaction. GST‐FOXM1D pulled down HA‐PKM2 expressed in 293T cells, which was determined by both anti‐HA and anti‐PKM2 antibodies (Fig. 1C).

To explore the precise binding region between FOXM1D and PKM2, we designed truncated mutants based on the structural domain of PKM2 (Fig. 1D) [33] and further expressed them with HA tag in 293T cells (Fig. 1E). The result of a Co‐IP assay using anti‐HA antibody demonstrated that PKM2 mutants A, B, and C, as well as intact PKM2, could capture the co‐expressed Flag‐FOXM1D (Fig. 1E), indicating that the domains A1 and/or B of PKM2 are most likely the binding site for FOXM1D. We further identified the precise binding region in PKM2 using the GST pull‐down assay. As shown in Fig. 1F, GST‐FOXM1D bound to the B but not the A1 domain of PKM2, as expressed in 293T cells. In addition, we also designed truncated mutants of FOXM1D that were based on the coding exons due to absence of the FOXM1 crystal structure (Fig. 1G) [28]. We expressed these with Flag tag in 293T cells, and using the Co‐IP assay with the anti‐Flag antibody, we mapped the binding mutants of FOXM1D to the co‐expressed PKM2 as mutants A, B, and D (Fig. 1H). Given that exons II and III of *FOXM1D* are shared by *FOXM1A*/*B*/*C/D* [28], we thus detected whether or not FOXM1A/B/C could physically bind to PKM2. We cotransfected 293T cells with plasmid expressing HA‐PKM2, Flag‐FOXM1A/B/C/D, or empty vector, then performed a Co‐IP assay using anti‐Flag antibody. As we expected, all of the four Flag‐tagged FOXM1 isoforms bound to HA‐PKM2 (Fig. S1A) and exhibited little effect on the transcription of PKM2 (Fig. S1B). Our findings suggested that exons II and III of *FOXM1D* encoded the binding region for PKM2, therefore clearly demonstrated the interaction between FOXM1D and PKM2.

### 3.2 FOXM1D promotes tumor aerobic glycolysis

**Fig. 2:**
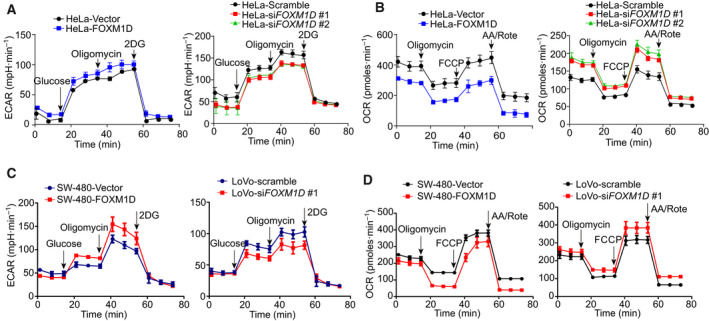
FOXM1D promotes glycolysis. (A, B) ECAR (A) and OCR (B) assays in HeLa cells stably overexpressing FOXM1D and vector control (left panel) and transfected with siRNA specific against FOXM1D or scramble siRNA (right panel). (C) and (D) ECAR (C) and OCR (D) assays in SW‐480 cells stably overexpressing FOXM1D or vector control, and in LoVo cells transfected with siRNA specific against FOXM1D or scramble siRNA. The ectopic expression of FOXM1D enhanced glycolytic activity, while its impaired mitochondrial function. In contrast, the insufficiency of FOXM1D played the opposite role.

PKM2 catalyzes the rate‐limiting step of aerobic glycolysis, and it plays a crucial role in the regulation of the Warburg effect [4, 34]. As FOXM1D can interact with PKM2, we sought to detect the effect of ectopic expression and knockdown FOXM1D on glycolysis by measuring the extracellular acidification rate (ECAR) and oxygen consumption rate (OCR) in HeLa overexpression FOXM1D cells. Ectopic expression of FOXM1D induced an appreciable increase, while FOXM1D knockdown led to a decrease, in ECAR, which reflects overall glycolytic flux (Fig. 2A), along with a decrease in OCR, which is an indicator of mitochondrial respiration (Fig. 2B). As FOXM1A/B/C all have an interaction with PKM2, we also measured ECAR and OCR in HeLa ectopic expression FOXM1A/B/C (Fig. S2A–F). FOXM1A/B/C failed to induce an obvious alteration in either ECAR or OCR when compared to the vector control. Considering that LoVo and SW‐480 cells display the highest and lowest expression levels of FOXM1D, respectively, in the six examined colorectal cell lines [28], we further compare the effect of FOXM1D on glycolysis between them. We measured the ECAR and OCR in SW‐480 cells that overexpressed FOXM1D and in LoVo cells where FOXM1D was knocked down by specific siRNA. We found that ectopic FOXM1D resulted in a notable elevation in the ECAR, whereas insufficient FOXM1D resulted in an ECAR reduction (Fig. 2C). In contrast, ectopic FOXM1D yielded a significant reduction in the OCR, while insufficient FOXM1D resulted in its elevation (Fig. 2D). Together, these results demonstrated that FOXM1D could effectively promote the Warburg effect.

### 3.3 FOXM1D inhibits PK activity of PKM2 tetramer by assembling a heterooctamer

**Fig. 3:**
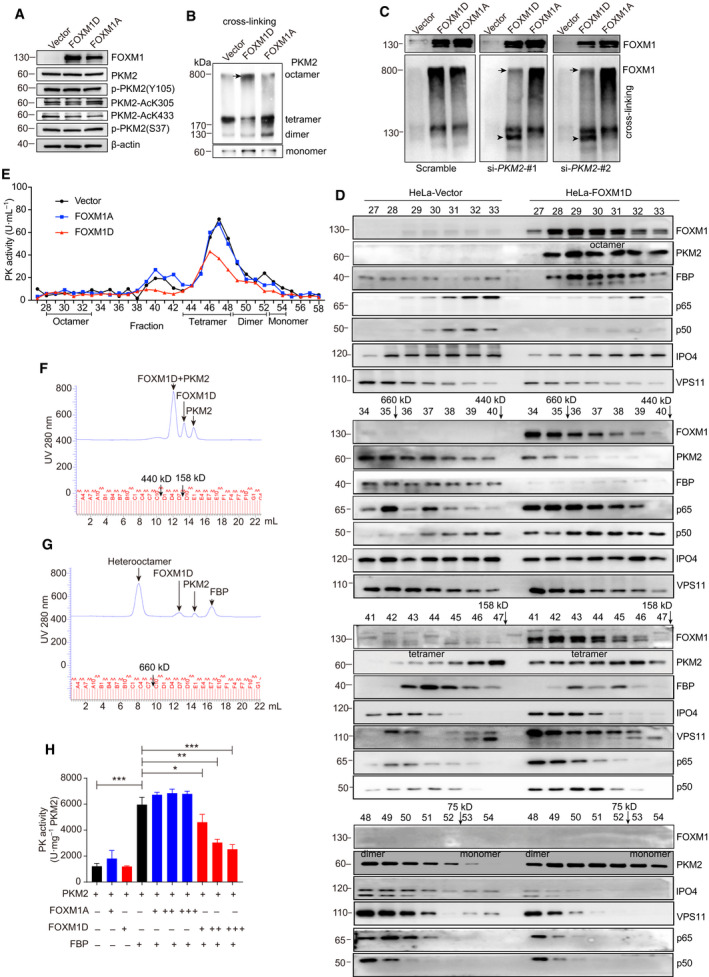
Tetrameric PKM2 assembles a heterooctamer with FOXM1D, thus reducing PK activity. (A) The phosphorylation and acetylation levels of PKM2 in HeLa cells with ectopic expression of FOXM1A/D. (B) Immunoassays detected that ectopic FOXM1D, but not FOXM1A, induced the emergence of a macromolecular protein complex (black arrow) containing PKM2 in cross‐linked samples from HeLa cells, thus altering the composition of PKM2 monomer and polymers. (C) The effect of PKM2 insufficiency on the formation of the FOXM1D and PKM2 complex. Up panel: the ectopic expression of FOXM1A/D using regular samples. Bottom panel: PKM2 insufficiency by specific siRNAs using cross‐linking samples reduced the FOXM1D‐containing (black arrow), but not the FOXM1A‐containing complex, leading to a new FOXM1 band (arrowhead). (D) Identification of the potential complex of FOXM1D and PKM2 in HeLa cells with ectopic expression of FOXM1D by gel chromatography and immunoblotting. FOXM1D assembles a heterooctamer with PKM2, predicted by the molecular weight. The fraction numbers, elution of molecular weight markers with arrows, and PKM2 monomer/polymers are indicated. (E) The PK activity of individual fractions from HeLa cells that were ectopic expression with vector, FOXM1A, or FOXM1D. (F) and (G) Identification of the interaction between FOXM1D and PKM2 in the absence (F) and presence (G) of FBP by gel chromatography. The three purified proteins were loaded in equal amounts (60 μg). (H) The measurement of PK activity in the different indicated mixtures. Each ‘+’ represents 10 μg of protein; thus, 10 μg of FOXM1/10 μg PKM2, 20 μg FOXM1/10 μg PKM2, and 30 μg FOXM1/10 μg PKM2 are about 0.665, 1.33, and 2, respectively, in molar ration. The data are analyzed by Student's *t*‐test (two‐tailed) and presented as mean ± SD; *n* = 3. *P < 0.05; **P < 0.01; and ****P < 0.0001 vs. control.

It has been reported that PK activity of PKM2 may be regulated by its post‐translation modification (PTM). Phosphorylation at Y105 may inhibit the formation of the active PKM2 tetrameter [35], acetylation at K305 may increase PKM2 degradation [13], and acetylation at K433 may interfere with FBP binding [7]. Considering that FOXM1A/D, but not FOXM1B/C, significantly regulate glycolysis without changing the PKM2 expression level as described previously, we first detected the PTM status of PKM2 by immunoblotting in HeLa cells with ectopic FOXM1A/D expression. The results showed that the levels of phospho‐Y105, acetyl‐K305, and acetyl‐K433 unchanged after ectopic FOXM1A/D expression (Fig. 3A), indicating that another mechanism was involved in the regulation of glycolysis. In addition, we found that ectopic FOXM1A/D expression failed to change the level of phospho‐S37 of PKM2, which is associated with nuclear translocation [18] (Fig. 3A).

The PK activity of PKM2 is also allosterically activated by fructose‐1,6‐biphosphate (FBP), whose binding to PKM2 causes the switch from a less active monomeric/dimeric form to an active tetrameric from [14, 36, 37]. Next, we determined the monomer: dimer: tetramer equilibrium of PKM2 using cross‐linking experiments with glutaraldehyde in HeLa cells with ectopic FOXM1A/D expression. Following glutaraldehyde cross‐linking, the proportion of the tetramer in FOXM1D‐overexpressing cells was notably reduced, while the monomer increased when compared with FOXM1A‐overexpressing and control cells (Fig. 3B). Interestingly, we observed that an obvious band ran close to the location of stacking gel (arrow in Fig. 3B). To understand whether or not this macromolecular band was associated with the complex of PKM2 and FOXM1D, we induced PKM2 insufficiency (Fig. 5J) by using two specific siRNAs in HeLa cells with ectopic FOXM1A/D (up panel in Fig. 3C). Immunoblotting assays using anti‐FOXM1 antibody revealed the presence of another notable band, other than a normal FOXM1 band, that was also located close to the stacking gel in the cell extracts treated with glutaraldehyde. This band was present in both the scramble siRNA‐treated HeLa cells (left bottom panel in Fig. 3C). However, PKM2 insufficiency provoked this band much weaker only in FOXM1D‐ but not FOXM1A‐overexpressing cells compared to scramble control, thus resulting in a dissociated new band close to the normal FOXM1 (middle and right bottom panels in Fig. 3C). These results suggested that FOXM1D, but not FOXM1A, could form a large heterogeneous polymer with PKM2 to regulate glycolysis.

In order to determine the size and composition of the heterogeneous polymer, we used gel chromatography to separate FOXM1D and PKM2 in the extract of FOXM1A/D‐overexpressing HeLa cells. The result of the immunoblotting assay for each fraction demonstrated that FOXM1D, PKM2, and FBP appeared simultaneously in fractions 28–35, whose size was a bit greater than the 660 kD marker in the fraction between 35 and 36 (Fig. 3D). The four B domains of the PKM2 tetramer were located at the outside corner [38], and the binding site of FOXM1D on PKM2 was located in the B domain (Fig. 1F), both of which may favor the binding of four FOXM1D molecules to the PKM2 tetramer without steric hindrance. Therefore, we hypothesized that this heterogeneous polymer was most likely a heterogeneous octamer, that is, a complex of four PKM2 (~ 60 kD), four FBP (~ 40 kD), and four FOXM1D (apparent ~ 130 kD). In addition, we observed that ectopic FOXM1D decreased the proportion of PKM2 tetramer accompanied by the reduced FBP (fractions 43–47), and increased those of the PKM2 dimer (fractions 48–52) and monomer (fractions 53–54) when compared with the vector control (Fig. 3D). It is notable that FOXM1A failed to form such a heterogeneous octamer with PKM2 in fractions 28–35 (Fig. S3), indicating that this physical interaction may be unstable. In addition, ectopic FOXM1A seemed to result in a slight increase in the proportion of PKM2 tetramer, accompanied by an increase in FBP (fractions 43–47) and PKM2 dimer (fractions 48–52) and a reduction in PKM2 monomer (fractions 53–54) (Fig. S3).

Given that the PKM2 tetramer displayed high PK activity, we next measured the enzyme activity of each fraction. The results showed that the heterogeneous octamer, which existed in fractions 28–33 in the extracts of FOXM1D‐overexpressing HeLa cell, showed undetectable PK activity (Fig. 3E). The PK activity of fractions 43–52, which contained PKM2 tetramer and dimer, decreased in the extracts of FOXM1D‐overexpressing compared with FOXM1A‐overexpressing and control HeLa cells (Fig. 3E). Interestingly, we found that fractions 38–42 from the extracts of the FOXM1A‐overexpressing and control, but not FOXM1D‐overexpressing HeLa cell displayed notable PK activity (Fig. 3E). This PK activity may result from the complex of PKM2 tetramer and other proteins, as FBP existed in these fractions and FOXM1D may have disrupted this complex (Fig. 3D).

To further confirm the physical interaction between FOXM1A/D and PKM2, we expressed and purified recombinant Flag‐FOXM1A/D and Flag‐FBP, and purchased PKM2. The results of SDS/PAGE (Fig. S4A) and gel chromatography (Fig. S4B–E) demonstrated the high purity and the principal existence form of the monomer. In the presence of excess FBP, the tetramer is the predominant form of PKM2, with trivial amount of monomer and without the dimer forms (Fig. S4F). However, in the absence of FBP but the presence of FOXM1A or FOXM1D, PKM2 formed a heterodimer with FOXM1A (Fig. S4G) or FOXM1D (Fig. 3F). Furthermore, in the presence of both FBP and FOXM1A, PKM2 preferred to form a homologous tetramer exclusive of binding to FOXM1A, although we did observe a tiny heterooctamer curve (Fig. S4H). In contrast, in the presence of FBP and FOXM1D, all of the PKM2 tetramer further bound to the four molecules of FOXM1D, leading to the assembly of a heterooctamer (Fig. 3G). This finding indicated that FOXM1D and FOXM1A displayed different binding activities to PKM2 tetramer. In the functional test, supplement of FBP alone, but not FOXM1A or FOXM1D alone, elevated the PK activity of PKM2 remarkably (Fig. 3H). The addition of FOXM1A weakly elevated the above FBP‐induced PK activity of PKM2 but without statistical significance; however, the addition of FOXM1D significantly reduced this PK activity in a dose‐dependent manner (Fig. 3H). Moreover, when providing excessive FOXM1D (1.33‐fold of PKM2 in molar ratio), about half (49%) of the PK activity of PKM2 was abolished. Therefore, these findings reveal that FOXM1D binds to the PKM2 tetramer to assemble a heterogeneous octamer, thereby reducing PK activity by about a half.

### 3.4 FOXM1D promotes tumor angiogenesis

**Fig. 4:**
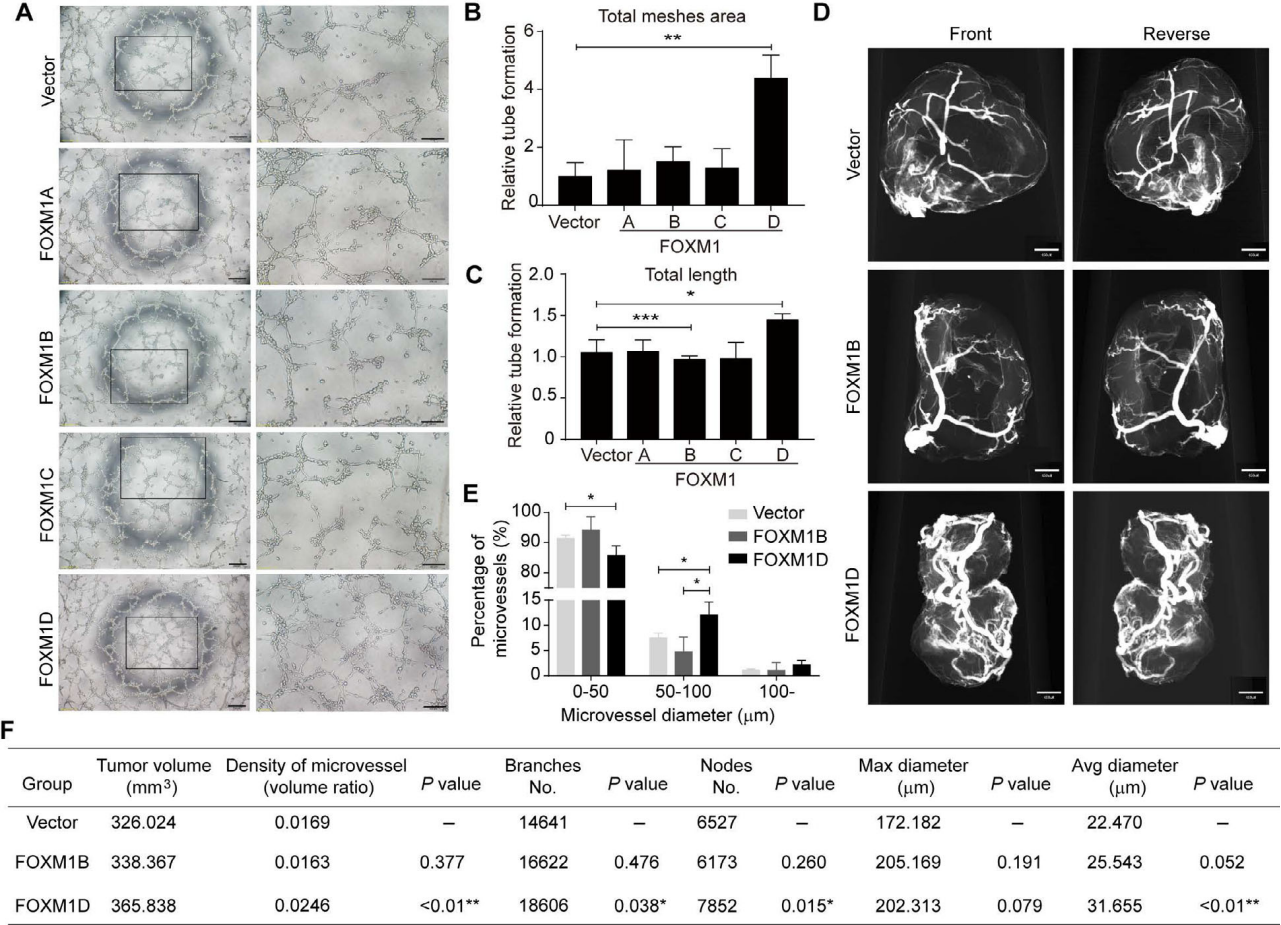
FOXM1D promotes angiogenesis. (A–C) The supernatant of cells with ectopic expression of FOXM1D, but not of FOXM1A/B/C, promoted HUVEC tube formation. The representative images (A) and the quantitative results of total meshes' area (B) and total length (C). Scale bar, 200 μm (left panel in A) or 100 μm (right panel in A). (D) The representative images of tumor blood vessels in the mice bearing HeLa cells stably overexpressing control, FOXM1B, or FOXM1D achieved by a microangiographic technique. Scale bar, 450 μm. (E) The quantitative results of microvessel diameter assessment. Three groups were divided because of the diameter. (F) The quantification of microvessel density, branch, nodes, maximal diameter, and average diameter in tumors (d). The data are analyzed by Student's *t*‐test (two‐tailed) and presented as mean ± SD; *n* = 3. *P < 0.05, **P < 0.01, and ***P < 0.001.

It has been recognized that the Warburg effect is closely associated with tumor angiogenesis [39, 40, 41], however, the underlying mechanisms remain elusive. Therefore, we detected the effect of ectopic FOXM1D expression on tumor angiogenesis. We collected the supernatant of FOXM1A/B/C/D‐overexpressing or control HeLa cells, and then used those to treat HUVECs before detecting the ability for tube formation. The results showed that ectopic expression of only FOXM1D, but not of FOXM1A/B/C and control significantly enhanced angiogenic sprouting and tube formation in HUVECs (Fig. 4A–C). This finding indicates that the supernatant of FOXM1D‐overexpressing HeLa cells may contain pro‐angiogenesis factor(s).

To verify this angiogenic effect *in vivo*, we qualified the tumor blood vessels by microangiography [42]. Considering that FOXM1B accelerates tumor growth more potently than FOXM1D [28], we selected tumor tissues of similar sizes to compare their microvessels. These tumor tissues were collected from previously generated tumor‐bearing mice that were implanted with vector control, FOXM1B‐, or FOXM1D‐overexpressing HeLa cells [28]. The images of tumor blood vessels show that ectopic FOXM1D, but not FOXM1B visibly enhanced angiogenesis compared with vector control (Fig. 4D). Furthermore, the quantitative results revealed that, although the tumor sizes were comparable in each group, the density, branches, nodes, maximal diameter, and average diameter of the microvessels in FOXM1D‐, but not FOXM1B‐overexpressing tumors were significantly greater than those of vector control tumors (Fig. 4E,F). Together, these results suggested that FOXM1D may strongly promote tumor neovascularization, most likely through releasing the pro‐angiogenesis factor, and more importantly, that the ample number of blood vessels induced by ectopic FOXM1D expression failed to accelerate tumor growth synchronously.

### 3.5 FOXM1D upregulates VEGFA expression mediated by PKM2 and NF‐κB

**Fig. 5:**
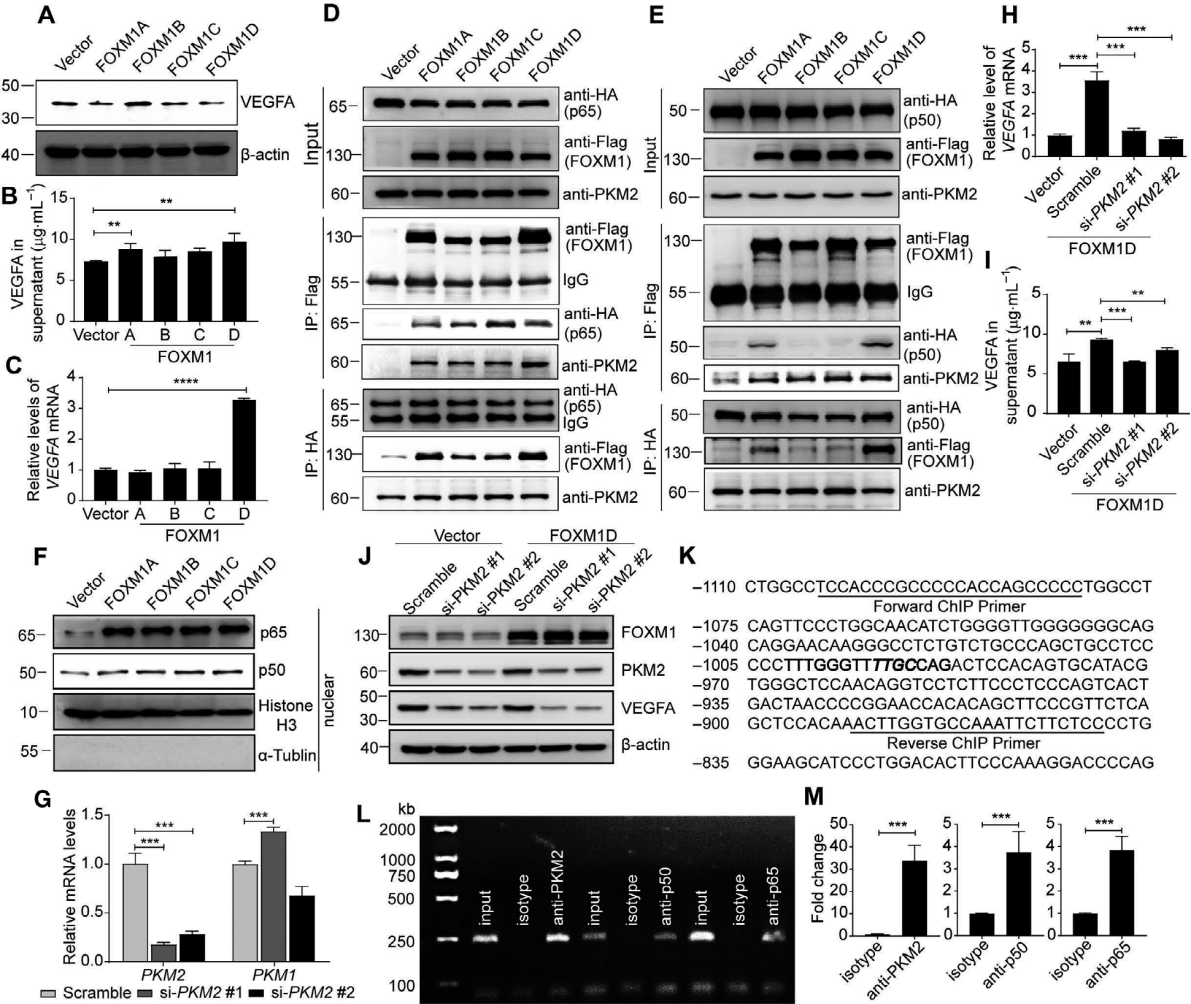
FOXM1D increases VEGFA expression by inducing the nuclear translocation of PKM2 and NF‐κB. (A, B) VEGFA protein levels in the total lysates (A) and supernatant (B) of HeLa cells with ectopic expression of FOXM1A/B/C/D. (C) *VEGFA* mRNA levels in HeLa cells with ectopic expression of FOXM1A/B/C/D. (D, E) The interaction among FOXM1A/B/C/D, NF‐κB (p65 in (D) or p50 in (E)), and PKM2. FOXM1A/B/C/D bound to PKM2 and p65 (D), while only FOXM1A, and especially FOXM1D, bound to p50 (E). (F) FOXM1A/B/C/D enhanced p65 and p50 nuclear translocation. (G) The mRNA levels of PKM1/2 after PKM2‐specific siRNA treatment. Their protein levels in cell lysate are shown in (J). (H–J) Insufficiency of PKM2 via siRNAs abrogated the FOXM1D‐induced increase in *VEGFA* in mRNA (H) and protein (supernatant in (I) and cell lysate in (J)) levels in HeLa cells. Data represent the mean ± SD from three independent experiments. (K–M) The specific antibody against PKM2, p50, or p65 could capture the fragment containing the same response element in the *VEGFA* promoter region (K). The binding site was predicted in bold sequences using matinspector software (Genomatix, Munich, Germany), in which the critical binding site was further highlighted in italic, and was amplified by the same specific primers (Table S2) using PCR in the ChIP assay (L). The quantitative data are also shown (M). The data are analyzed by Student's *t*‐test (two‐tailed) and presented as mean ± SD; *n* = 3. **P < 0.01, ***P < 0.001, and ****P < 0.0001 *vs*. control.

VEGF/VEGF‐receptor signaling has been well‐established as a key mediator of tumor angiogenesis [43]. Therefore, to understand the molecular mechanism of the FOXM1D angiogenic effect and its relationship with PKM2‐mediated glycolysis, we first detected the expression level of VEGFA in the cell lysate of FOXM1D‐overexpressing HeLa cells. Unexpectedly, ectopic FOXM1D even reduced the VEGFA level compared with vector control (Fig. 5A). However, we observed that VEGFA levels were significantly increased in the supernatants of FOXM1A/D‐ but not of FOXM1B/C‐overexpressing HeLa cells (Fig. 5B). This result indicated that, in FOXM1D‐overexpressing HeLa cells, VEGFA was most likely secreted extracellularly. Although FOXM1B has been reported to increase *VEGF* transcription in glioma cells [44], we observed that only FOXM1D, but not FOXM1A/B/C caused a marked upregulation in *VEGFA* transcription as detected by quantitative real‐time PCR (qRT**–**PCR) (Fig. 5C).

**Fig. 6:**
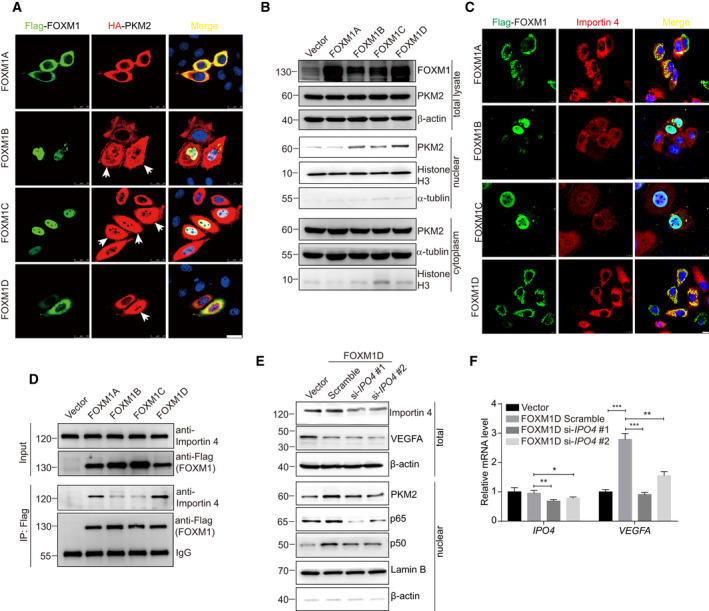
Importin 4 mediates FOXM1D‐induced nuclear translocation of PKM2 and NF‐κB and the resultant VEGFA upregulation via interaction with FOXM1D. (A) The subcellular colocalization of Flag‐FOXM1A/B/C/D and HA‐PKM2. The nuclear translocation of PKM2 increased upon the ectopic expression of FOXM1B/C/D, but not of FOXM1A, as determined by ICC assay in HeLa cells (white arrows). The interaction of FOXM1A/D with PKM2 was predominantly located in the cytoplasm, while that of FOXM1B/C with PKM2 existed mainly in the nucleus. Scale bar, 25 μm. (B) The subcellular detection of PKM2 by immunoblotting in HeLa cells with the ectopic expression of FOXM1A/B/C/D. The ectopic expression of FOXM1A/B/C/D failed to alter the PKM2 level (up panel in B). However, the ectopic expression of FOXM1B/C/D, but not of FOXM1A, promoted the nuclear translocation of PKM2 (middle panel in (B)) along with a slight decrease in cytoplasmic PKM2 (bottom panel in (B)). (C, D) FOXM1A/D, but not FOXM1B/C, bound strongly to importin 4, as determined by ICC (C) and Co‐IP (D) assays. Scale bar, 10 μm. (E) Insufficiency of importin 4 induced by specific siRNAs abrogated the effect of FOXM1D on the promotion of *VEGFA* transcription and suppresses FOXM1D‐induced nuclear translocation of PKM2 and NF‐κB. (F) Insufficiency of importin 4 thus further reduced VEGFA levels in the total lysate. The data are analyzed by Student's *t*‐test (two‐tailed) and presented as mean ± SD; *n* = 3. *P < 0.05; **P < 0.01; and ***P < 0.001 *vs*. control.

Considering the facts that: (a) nuclear PKM2‐controlled HIF‐1α and NF‐κB regulate *VEGFA* transcription in hypoxic conditions [22]; (b) nuclear PKM2 promotes angiogenesis by directly interacting with the NF‐κB p65 subunit [23]; (c) FOXM1 directly interacts with the NF‐κB subunit p65 [45]; and (d) FOXM1D, PKM2, and p65/p50 co‐existed in fractions 34–36 in the gel chromatography experiment (Fig. 3D), we therefore hypothesized that FOXM1D may promote *VEGFA* transcription by interacting with PKM2 and NF‐κB. Although we previously failed to identify any NF‐κB subunits as FOXM1D‐binding proteins by Co‐IP and LC/MS analysis (Data S1), we still conducted the Co‐IP assay in order to detect the interaction between FOXM1A/B/C/D and NF‐κB. In the vector control and Flag‐FOXM1A/B/C/D‐overexpressing HeLa cells, we further transfected HA‐p65‐ or HA‐p50‐expressing plasmid. The results of the Co‐IP assay revealed that anti‐Flag antibody captured HA‐tagged p65 and endogenous PKM2 in all of the FOXM1A/B/C/D‐overexpressing cells (Fig. 5D,E), while it could capture HA‐tagged p50 only in Flag‐FOXM1A‐, and especially Flag‐FOXM1D‐overexpressing cells (Fig. 5E). In addition, anti‐HA antibody captured Flag‐tagged FOXM1A/B/C/D in HA‐p65‐overexpressing cells (Fig. 5D) and endogenous PKM2 in either HA‐p65‐ or HA‐p50‐overexpressing cells (Fig. 5D,E); however, anti‐HA antibody bound only slightly to Flag‐FOXM1A, but bound strongly to Flag‐FOXM1D (Fig. 5E). Both nuclear subunits p65 and p50 increased simultaneously in HeLa cells with ectopic expression of FOXM1A/B/C/D (Fig. 5F, and Fig. S5A–C), resulting in the reduced levels of p65 and p50 in cytoplasm but not in total cell lysate (Fig. S5D). In addition, FOXM1A promotes the nuclear entry of NF‐κB but not PKM2 (Figs 5F and 6A, and Fig. S5A–C), and thus, there is no NF‐κB and PKM2 transcriptional complex in nucleus. FOXM1B/C promotes the nuclear entry of both NF‐kB and PKM2 (Figs 5F and 6A, and Fig. S5A–C); However, FOXM1B/C also enter the nucleus to disrupt the formation of NF‐κB/PKM2 complex. The nuclear PKM2 prefers to bind to FOXM1B/C (Fig. 6A) but not to NF‐κB (Fig. S5C), indicating that there is also no NF‐κB and PKM2 complex in nucleus. In contrast, FOXM1D also promotes the nuclear entry of both NF‐κB and PKM2 (Figs 5F and 6A, and Fig. S5A–C), while FOXM1D still stays in cytoplasm (Fig. 6A, and Fig. S5A,B). Therefore, the nuclear PKM2 can assemble a complex with NF‐κB in nucleus (Fig. S5C) without the disturbance of FOXM1D. These results suggest that only FOXM1D, but not FOXM1A/B/C, may upregulate *VEGFA* transcription by simultaneously promoting the nuclear translocation of NF‐κB and PKM2, and further assembling the NF‐κB and PKM2 transcriptional complex.

Next, we detected the effect of PKM2, the critical regulator in glycolysis, on FOXM1D‐induced *VEGFA* transcription. Two specific siRNAs against PKM2 reduced *PKM2* mRNA levels. However, si‐PKM2 #1 induced the restoration of *PKM1* mRNA, while si‐PKM2 #2 downregulated *PKM1* transcription (Fig. 5G). We further observed that the PKM2 insufficiency induced by two siRNAs almost completely abrogated FOXM1D‐induced *VEGFA* transcription (Fig. 5H), secretion (Fig. 5I), and translation (Fig. 5J) in FOXM1D‐overexpressing cells. Therefore, PKM2 may effectively regulate VEGFA expression independent of PKM1.

To confirm that PKM2 and NF‐κB may regulate *VEGFA* transcription as transcriptional factors, we conducted a ChIP assay in FOXM1D‐overexpressing HeLa cells in order to identify whether or not PKM2 and NF‐κB bind directly to the same *VEGFA* promoter region. The specific antibody against PKM2, p50, or p65, but not isotype IgG enriched this promoter fragment remarkably (Fig. 5K–M), which was amplified by PCR with the same primer pair (Fig. 5K). We further knocked down the expression of PKM2 or p65 to verify the binding specificity. Insufficiency of PKM2 significantly impaired the capability of enriching the *VEGFA* promoter fragment not only by PKM2 antibody but also by p65 or p50 antibody, and insufficiency of NF‐κB subunit p65 also displayed the similar results (Fig. S6A–D). Together, these results revealed that FOXM1D binds to PKM2 and the NF‐κB subunits p65 and p50, further promotes their nuclear translocation, thus leading to increased VEGFA expression as regulated by PKM2 and NF‐κB transcriptional complex.

### 3.6 The binding of FOXM1D to importin 4 promotes the nuclear translocation of PKM2 and NF‐κB

FOXM1D locates predominantly in the cytoplasm, while PKM2 and NF‐κB translocate into nucleus to regulate *VEGFA* transcription upon ectopic FOXM1D. Therefore, we further explored how FOXM1D promotes nuclear translocation of PKM2 and NF‐κB. The result of Co‐IP analysis demonstrated the interaction between FOXM1A/B/CD and PKM2 (Fig. S1A). Next, we employed ICC analysis to verify their localization. As reported previously [28], FOXM1B/C locate predominantly in the nucleus while FOXM1A/D locate predominantly in the cytoplasm (Fig. 6A). Ectopic expression of all four FOXM1 isoforms did not alter the expression level of PKM2, while ectopic expression of FOXM1B/C/D, but not FOXM1A, promoted the nuclear translocation of PKM2 (Fig. 6A,B). Due to the higher nuclear PKM2 translocation, we thus observed a slight reduction in the level of PKM2 in the cytoplasm of FOXM1B/C/D‐ but not FOXM1A‐overexpressing cells (Fig. 6B).

Next, we interrogated the mechanism by which FOXM1D promotes PKM2 and NF‐κB translocation into nucleus. Although importin‐α/β are responsible for the import of many proteins from the cytoplasm to the nucleus, a host of importins related to β‐importins has been described to interact with the nuclear localization signal of specific proteins and import them into the nucleus independently of importin‐α. For example, importin‐4 transports vitamin D receptor [46] as well as transition protein 2 [47], and importin‐7 transports HIV‐1 intracellular reverse transcription complexes [48] into the nucleus. Interestingly, we observed that both importin 4 and importin 7 were captured by FOXM1D with anti‐Flag antibody (Data S1). Using Co‐IP and ICC assays, we demonstrated that importin 4 bound to FOXM1A/D but not to FOXM1B/C (Fig. 6C,D), indicating that FOXM1B/C employed a different approach to inducing nuclear translocation of PKM2 and NF‐κB from FOXM1A/D. In addition, results of the gel chromatography experiment indicated that importin 4 universally located in fractions of 34–40 (Fig. 3D), suggesting that it binds to a variety of proteins including PKM2 and NF‐κB. Furthermore, insufficiency of importin 4 induced by specific siRNAs abrogated the effect of ectopic FOXM1D to increase *VEGFA* transcription, which most likely resulted from the subsequently reduced nuclear levels of PKM2 and NF‐κB (Fig. 6E,F). We also found that the levels of VEGFA in total lysate (Fig. 6E) decreased accordingly. These results suggest that FOXM1D promotes the nuclear translocation of PKM2 and that NF‐κB may be mediated by importin 4, thus leading to the upregulation of VEGFA.

### 3.7 FOXM1D promotes VEGFA release by interacting with VPS11

**Fig. 7:**
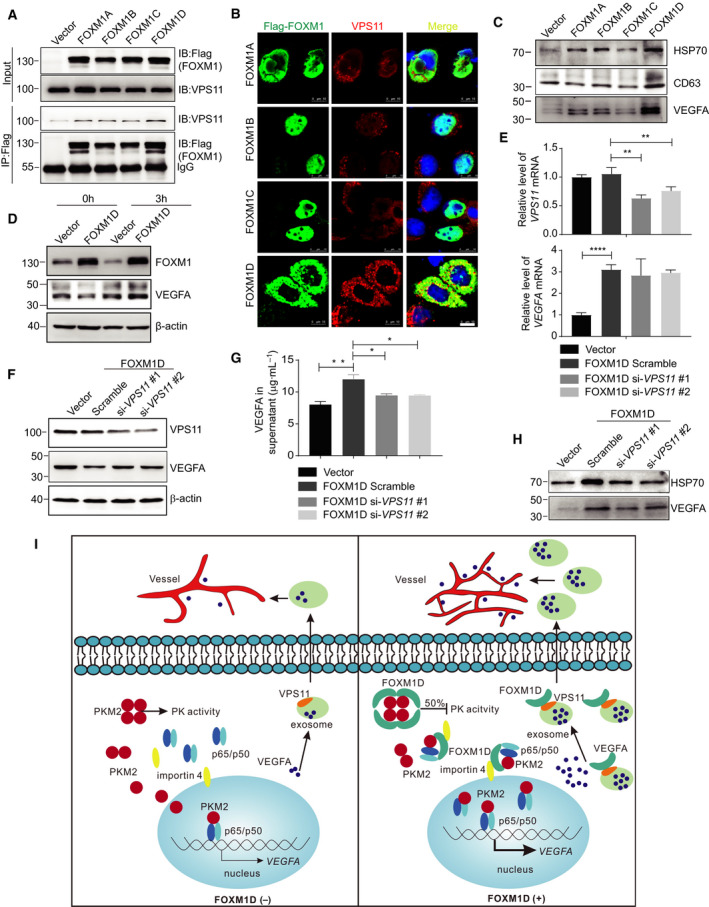
FOXM1D promotes the secretion of VEGFA‐containing exosomes by interacting with VPS11. (A, B) Flag‐FOXM1A/B/C/D all interacted with endogenous VPS11, as identified by Co‐IP (A) and ICC (B) assays. FOXM1D binding to VPS11 displayed stronger affinity than FOXM1A/B/C (A), and FOXM1A/D, but not FOXM1B/C, colocalized with VPS11 in the cytoplasm (B). Scale bar, 10 μm. (C) FOXM1D strongly promoted the secretion of VEGFA‐containing exosomes. A serum‐free medium was changed and cultured for 12h before collection. (D) The effect of monensin treatment (10 μm for 3 h) on VEGFA level in total cell lysate of vector control or FOXM1D‐overexpressing HeLa cells. (E) Insufficiency of VPS11 induced by specific siRNAs did not alter *VEGFA* transcription. (F–H) Insufficiency of VPS11 increased the accumulation of VEGFA in the total lysate (F), while it reduced VEGFA levels in the supernatant (G) and exosome (H). (I) Working model for FOXM1 potentiating PKM2‐mediated tumor glycolysis and angiogenesis. The data are analyzed by Student's *t*‐test (two‐tailed) and presented as mean ± SD; *n* = 3. *P < 0.05; **P < 0.01; and ****P < 0.0001 *vs*. control.

Our study found that ectopic expression of FOXM1D remarkably increased *VEGFA* transcription (Fig. 5C); however, the level of VEGFA protein decreased conversely in the cell lysate (Fig. 5A) and only slightly increased in the supernatant (Fig. 5B). The above evidence did not seem enough to support the potent pro‐angiogenic effect of FOXM1D (Fig. 4). We therefore interrogated the underlying mechanism for FOXM1D‐dependent VEGFA extracellular release. FOXM1 has been demonstrated to interact with HSP70, a critical exosome biomarker [49]. Using Co‐IP and LC/MS analysis, we previously identified that FOXM1D also interacted with VPS11 (Data S1), an exosome component [50]. It has been shown that VPS11 plays an important role in vesicle trafficking and fusion of lysosomes and endosomes [51]. Considering the importance of the exosome in angiogenesis [52, 53], we therefore hypothesized that FOXM1D might facilitate VEGFA release via the exosome by interacting with VPS11. Using a Co‐IP assay, we first verified that anti‐Flag antibody could capture the endogenous VPS11 in all of the Flag‐FOXM1A/B/C/D‐overexpressing HeLa cell extracts (Fig. 7A), indicating that FOXM1A/B/C/D interacted physically with VPS11 in the cell extracts. We also employed an ICC assay to detect the subcellular colocalization of FOXM1A/B/C/D and VPS11. Due to the distinct subcellular location of VPS11 and FOXM1B/C, we failed to observe their virtual interaction (Fig. 7B). In contrast, we did observe sporadic formation of FOXM1A, while formation of FOXM1D exhibited the massive dot‐like overlap with VPS11 (Fig. 7B). This finding suggested the strong involvement of FOXM1D in the formation of the exosome.

To understand the function of ectopic FOXM1D on regulating exosome and VEGFA release, we further collected the exosomes from the supernatant of vector control or FOXM1A/B/C/D‐overexpressing HeLa cells, in which the cell number and supernatant volumes were same. Ectopic FOXM1A/B/C slightly elevated, while FOXM1D strongly elevated the mount of exosomes in the supernatant, as determined by HSP70, CD63, and VEGFA levels (Fig. 7C). The Golgi inhibition by monensin led to more intracellular accumulation of VEGFA in the FOXM1D‐overexpressing cells compared with the vector control cells and FOXM1D‐overexpressing but no monensin pretreatment cells (Fig. 7D), indicating that FOXM1D promotes VEGFA release via exosome. In addition, FOXM1D insufficiency abrogated the ectopic VEGFA‐induced increase in VEGFA release via exosome (Fig. S7). Further, VPS11 insufficiency induced by specific siRNAs (upper panel in Fig. 7E,F) failed to alter the effect of ectopic FOXM1D on increasing *VEGFA* transcription (bottom panel in Fig. 7E). However, it is possible that VPS11 insufficiency may result in exosome dysfunction, thus reducing the level of VEGFA in the supernatant (Fig. 7G) and in exosome (Fig. 7H), while elevating VEGFA levels in total cell lysate (Fig. 7F). Therefore, these results suggested that FOXM1D interaction with VPS11 promoted exosome and VEGFA release.

## 4 Discussion

Adequate nutrients and rich vasculature are the prerequisites for the large tumor size associated with late‐stage cancer. During this process, PKM2 appears to play a convergent role through the regulation of glycometabolism, transcription, and angiogenesis. Herein, we have provided another mechanism by which PKM2 regulates this process. Through protein–protein interaction, the PKM2 tetramer assembles a heterooctamer with FOXM1D, thus significantly restraining its PK activity and oxidative phosphorylation to about 50% (Fig. 7I). In addition, PKM2 and NF‐κB were translocated together into the nucleus to regulate *VEGFA* transcription, which was mediated by the binding of FOXM1D to the PKM2 and NF‐κB complex and to the nuclear transporter importin 4. Furthermore, the increased VEGFA was secreted via the exosome to promote angiogenesis, which was interestingly mediated by another FOXM1D‐binding partner, VPS11, an important component of the exosome (Fig. 7I).

Among the four FOXM1 isoforms, nuclear‐located FOXM1B/C are the subject of much more investigation, as they are the primary transcription factors involved in proliferation‐related gene transcription. In addition, FOXM1B/C have been demonstrated to promote cancer progression through the interaction with other proteins, such as β‐catenin, STAT3, SMAD3, HSP70, nucleophosmin (NPM), maternal embryonic leucine‐zipper kinase (MELK), and Pin1 [54]. FOXM1D is predominantly located in the cytoplasm and is implicated in the remarkable upregulation that is seen in advanced colorectal cancer with liver metastasis, in which FOXM1D promotes EMT by interacting with and activating ROCKs [28]. By coincidence, an increase in PKM2 expression was also reported in late stage of colorectal cancer and was associated with metastasis [55]. Here, we further demonstrated that FOXM1D, acting as an adhesive protein, continues to interact with multiple proteins such as PKM2, the NF‐κB subunits of p65 and p50, importin 4, and VPS11, and thus promotes tumor glycolysis and angiogenesis. Unfortunately, the crystal structure of FOXM1 remains unknown, most likely due to its highly flexible conformation, which thus makes it prone to interacting with multiple proteins.

Metabolic reprogramming is fundamental to cancer initiation and progression, in which PKM2 has been demonstrated to play a central role [56]. PKM2 is upregulated in most types of cancer cells, and dynamically exchanges between its tetrameric and dimeric forms. Dimeric PKM2 exhibits less PK activity and may be the main form that exists in cancer cells, thereby leading to accumulation of the glycolytic intermediates upstream of PKM2 and further providing a high level of metabolic precursors for synthetic processes. Aside from its expression level, PKM2 enzyme activity is tightly regulated by posttranslational modification, allosteric metabolic intermediates, and protein–protein interaction [57, 58]. Phosphorylation at tyrosine 105 disrupts the formation of the tetramer form by inducing FBP release, thus decreasing PKM2 enzymatic activity [35]. Acetylation at lysine 305 or 433 decreases PKM2 enzymatic activity by distinct mechanisms [7, 13]. Both FBP and serine are effective allosteric activations of PKM2 through the stabilizing of PKM2 in its active tetramer conformation [58]. Succinyl‐5‐aminoimidazole‐4‐carboxamide‐1‐ribose‐5′‐phosphate (SAICAR), an intermediate of the de novo purine nucleotide biosynthesis pathway, can stimulate PKM2's activities of both pyruvate kinase and protein kinase [59, 60]. Mucin 1‐C [61] and death‐associated protein kinase (DAPK) [62] activate, while promyelocytic leukemia (PML) tumor‐suppressor protein [63] reduces the pyruvate kinase activity of PKM2 via protein–protein interaction. Herein, we revealed that the PKM2 domain B interacts directly with the encoded sequences of FOXM1 exons II and III, indicating that all four FOXM1 isoforms may bind to PKM2. Because PKM2 and FOXM1A/D, but not FOXM1B/C, mainly locate in the cytoplasm, we can postulate that the PK activity of PKM2 is probably regulated by FOXM1A/D. Indeed, FOXM1D causes a remarkable decrease in PKM2 enzymatic activity, while FOXM1A exerts the opposite effect. Interestingly, FOXM1 (B) has been demonstrated to promote the Warburg effect in pancreatic cancer by increasing LDHA transcription [64]. Moreover, FOXM1D, but not FOXM1A, assembles a heterooctamer with the PKM2 tetramer, therefore reducing the enzymatic activity of PKM2 by about a half, an effect that is probably due to the steric hindrance for the substrate binding. The enhanced Warburg effect mediated by FOXM1D may therefore promote cancer progression by increasing the production of metabolic precursors for synthetic processes.

PKM2 can also translocate into the nucleus and regulates gene expression, likely as dimer [14] or monomer [16, 56]. We further revealed that FOXM1D also strongly induces the nuclear translocation of PKM2 and NF‐κB via multiple protein–protein interactions, a process that was mediated by the interaction of FOXM1D with the soluble nuclear transport factor importin 4. The nuclear complex of PKM2 and NF‐κB then binds to the *VEGFA* promoter region, further increasing VEGFA expression. PKM2 insufficiency almost completely abrogates FOXM1D‐induced *VEGFA* upregulation. After secretion, VEGFA is mainly contained in the exosome rather than in the soluble supernatant. More interestingly, FOXM1D further interacts with VPS11 and most likely also interacts with HSP70 [65], resulting in the promotion of the assembly and release of exosomes that contain VEGFA. Eventually, FOXM1D‐induced VEGFA release in the exosome strongly promotes tumor angiogenesis and progression. It should be noted that when compared with the proliferation‐specific transcription factor FOXM1B [29, 66], FOXM1D induced significantly slower tumor growth in tumor‐bearing mice, although it could potently promote angiogenesis. This finding indicated that blood vessels might be not enough to support tumor growth, and therefore, anti‐angiogenic drugs alone might be not be sufficient to curb cancer establishment and growth.

FOXM1 plays a critical role in each of the steps of tumor initiation, progression, metastasis, and treatment [67, 68, 69]. It is thus being acknowledged in all hallmarks of cancer with various mechanisms [70]. FOXM1, referred to as FOXM1B probably and FOXM1C in most cases, has been identified to be strongly upregulated in almost all types of tumors and as a major predictor for cancer prognosis by gene landscape analysis [25, 26, 31, 71]. However, it seems challengeable to explain the importance of FOXM1 in cancer by only describing the ability of FOXM1 (FOXM1B/C) to regulate the transcription of over 200 target genes that are primarily involved in cell proliferation [29, 30]. Alternative splicing may diversify the functional characteristics of some genes even with minor variants of each other, thus contributing to the functional complexity that results from the diverse sequence and subcellular distribution on the protein level [72]. Herein, our findings have provided a new insight into the PKM2‐induced tumor glycolysis and angiogenesis that was potentiated by multiple protein–protein interactions mediated by FOXM1D, and suggest that FOXM1 may be a promising target for cancer therapeutics.

### 4.1 Quantification and statistical analysis

The statistics was performed with graphpad prism 7.0 (GraphPad Software, La Jolla, CA, USA). The results were presented as mean ± SD or mean ± SEM, as indicated in the figure legends. Differences in parametric data between two groups were evaluated by Student's *t*‐test (two‐tailed). A *P* value < 0.05 was considered significant, and the level of significance was indicated as ns, not statistically significant; *P < 0.05 and **P < 0.01, ***P < 0.001 *vs*. control.

## Conflict of interest

The authors declare no conflict of interest.

## Author contributions

WH conceived and supervised the project; WH and WZ wrote the manuscript; WZ, XZ, and SH performed most of the experiments and analyzed the data, with assistance from JC, PD, QW, Luying Li, XL, Ling Li, PZ, and DZ, in which XZ and SH performed, and WH and JW supervised the microangiography experiment. QYL and JW contributed ideas.

## Supporting information


**Fig. S1.** Identification of the interaction between FOXM1A/B/C/D and PKM2.
**Fig. S2.** FOXM1A/B/C have no effect on glycolysis.
**Fig. S3.** Gel filtration to verify the polymer formed by identification of the interaction between FOXM1A and PKM2.
**Fig. S4.** Gel filtration to verify the purified protein and polymer formation. Gel filtration to verify the purified protein and polymer formation.
**Fig. S5.** Identification of the interaction between FOXM1A/B/C/D and NF‐κB.
**Fig. S6.** FOXM1D Upregulates VEGFA Expression Mediated by PKM2 and NF‐κB.
**Fig. S7.** FOXM1D regulates VEGFA release via exosome.

**Table S1.** Information on antibodies used in the study.

**Table S2.** Information on primers and siRNA sequence used in the study.

**Data S1.** Anti FOXM1D‐CoIP‐LCMS.
