# Supplementary material for: FOXM1D potentiates PKM2‐mediated tumor glycolysis and angiogenesis
Source: Mol Oncol. 2021 Apr 2;15(5):1466–85. doi: 10.1002/1878-0261.12879 (PMC8096781; doi:10.1002/1878-0261.12879)
Supplement: Supplementary file 1 — Fig. S1. Identification of the interaction between FOXM1A/B/C/D and PKM2. Fig. S2. FOXM1A/B/C have no effect on glycolysis. Fig. S3. Gel filtration to verify the polymer formed by identification of the interaction between FOXM1A and PKM2. Fig. S4. Gel filtration to verify the purified protein and polymer formation. Gel filtration to verify the purified protein and polymer formation. Fig. S5. Identification of the interaction between FOXM1A/B/C/D and NF‐κB. Fig. S6. FOXM1D Upregulates VEGFA Expression Mediated by PKM2 and NF‐κB. Fig. S7. FOXM1D regulates VEGFA release via exosome. [file MOL2-15-1466-s004.pdf]

Supplemental Figures

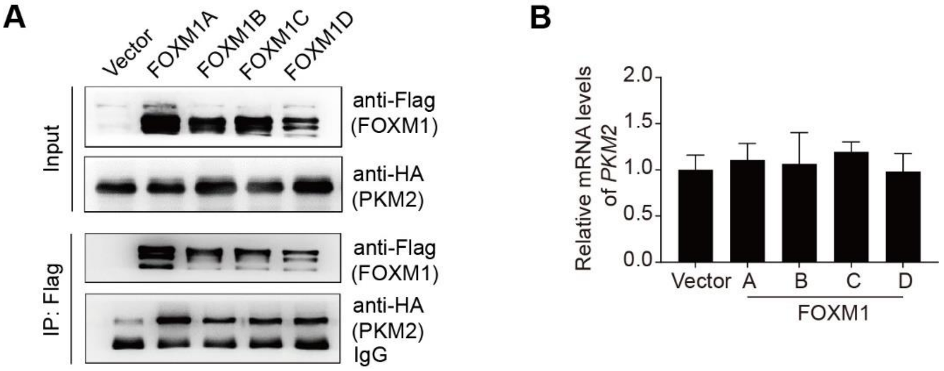

Fig. S1 Identification of the interaction between FOXM1A/B/C/D and PKM2. **A** The interaction between FOXM1A/B/C/D and PKM2. FOXM1A/B/C/D bound to PKM2. **B** The transcriptional level of PKM2 in HeLa over-expressing FOXM1A/B/C/D and Vector.

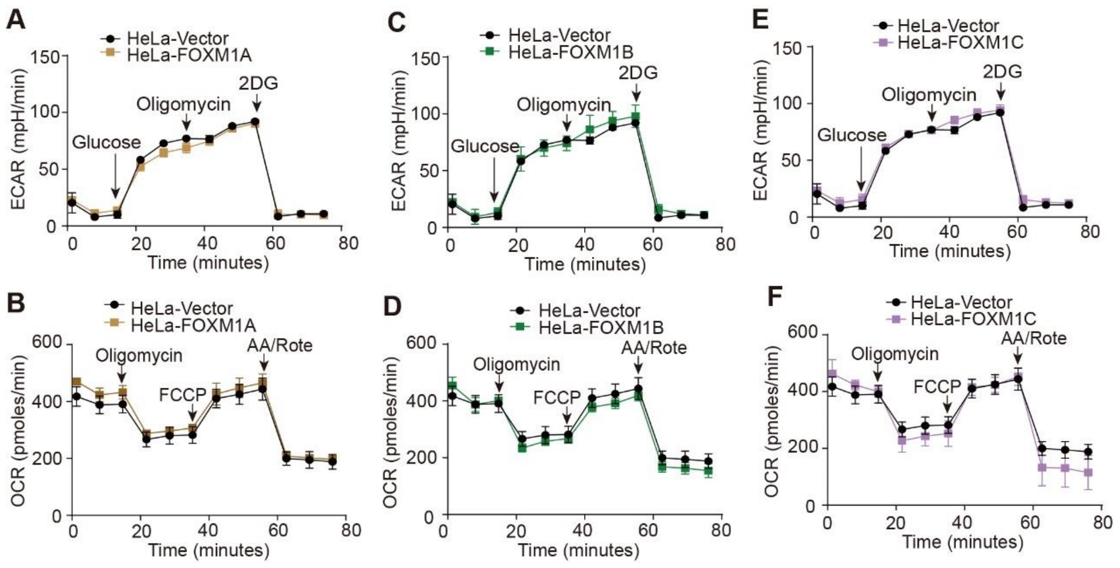

Fig. S2 FOXM1A/B/C have no effect on glycolysis. **A-F** The ectopic expression of FOXM1A (A-B), FOXM1B (C-D) or FOXM1C (E-F) failed to affect glycolysis in HeLa cells detected by ECAR (A, C and E) and OCR (B, D and F) compared to the vector control.

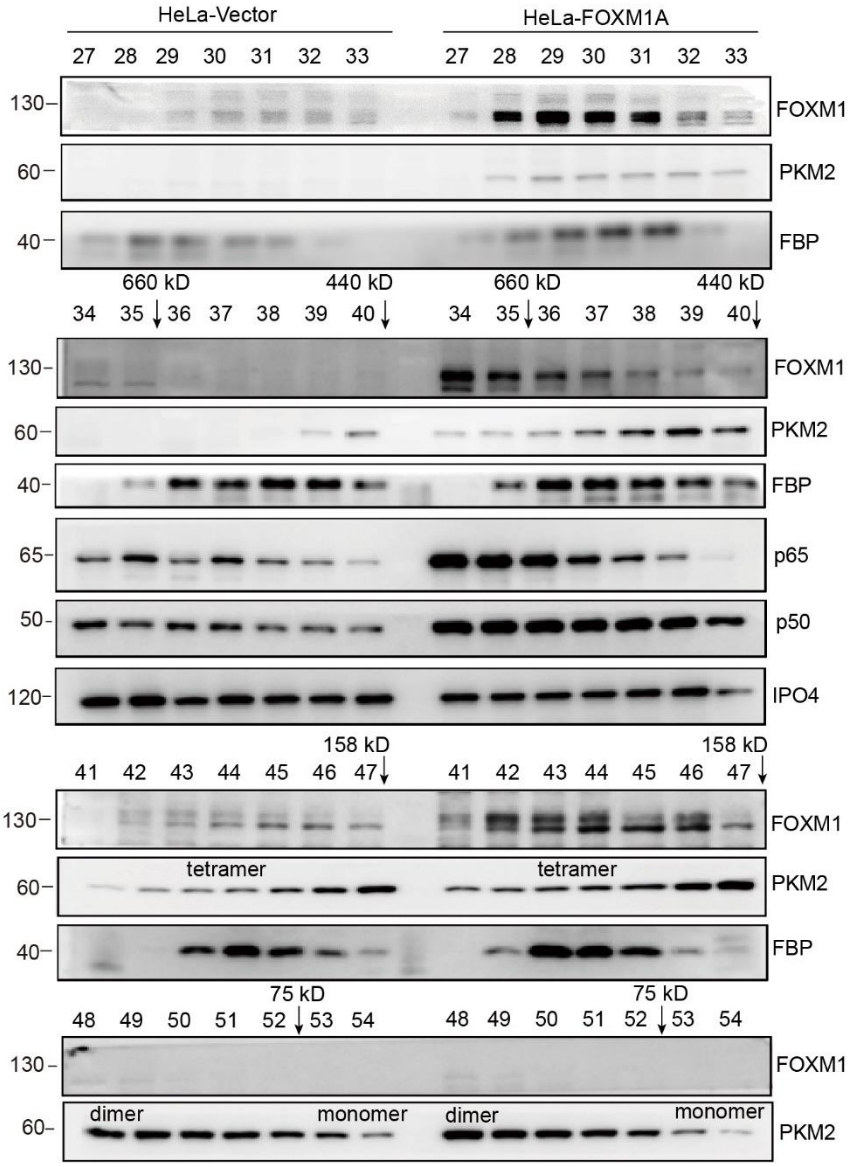

Fig. S3 Gel filtration to verify the polymer formed by identification of the interaction between FOXM1A and PKM2. Identification of the potential complex of FOXM1A and PKM2 in HeLa overexpressing FOXM1A cells by gel chromatography and immunoblotting. Fraction numbers, elution of molecular weight markers with arrows, and PKM2 monomer/polymers are indicated.

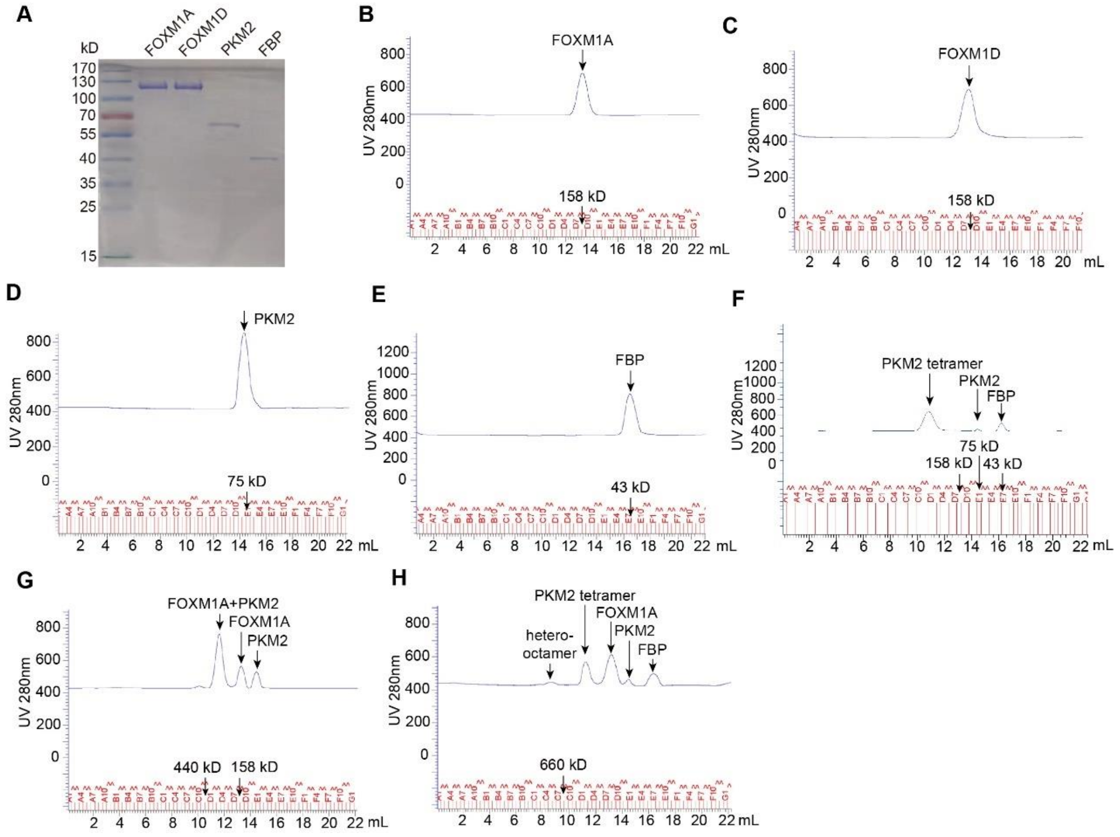

Fig. S4 Gel filtration to verify the purified protein and polymer formation. A The purity of the indicated proteins was detected by 10% SDS-PAGE and Coomassie bright blue staining. **B-E** The purified protein of FOXM1A (B), FOXM1D (C), PKM2 (D), and FBP1 (E). Fraction numbers and elution of molecular weight markers with arrows are indicated. **F-H** The equal weight of proteins PKM2 and FBP1 (F); FOXM1A and PKM2 (G); and FOXM1A, PKM2 and FBP1 (H) were mixed to form a complex and polymer formation was detected by gel filtration. Fraction numbers, elution of molecular weight markers with arrows, and polymers are indicated.

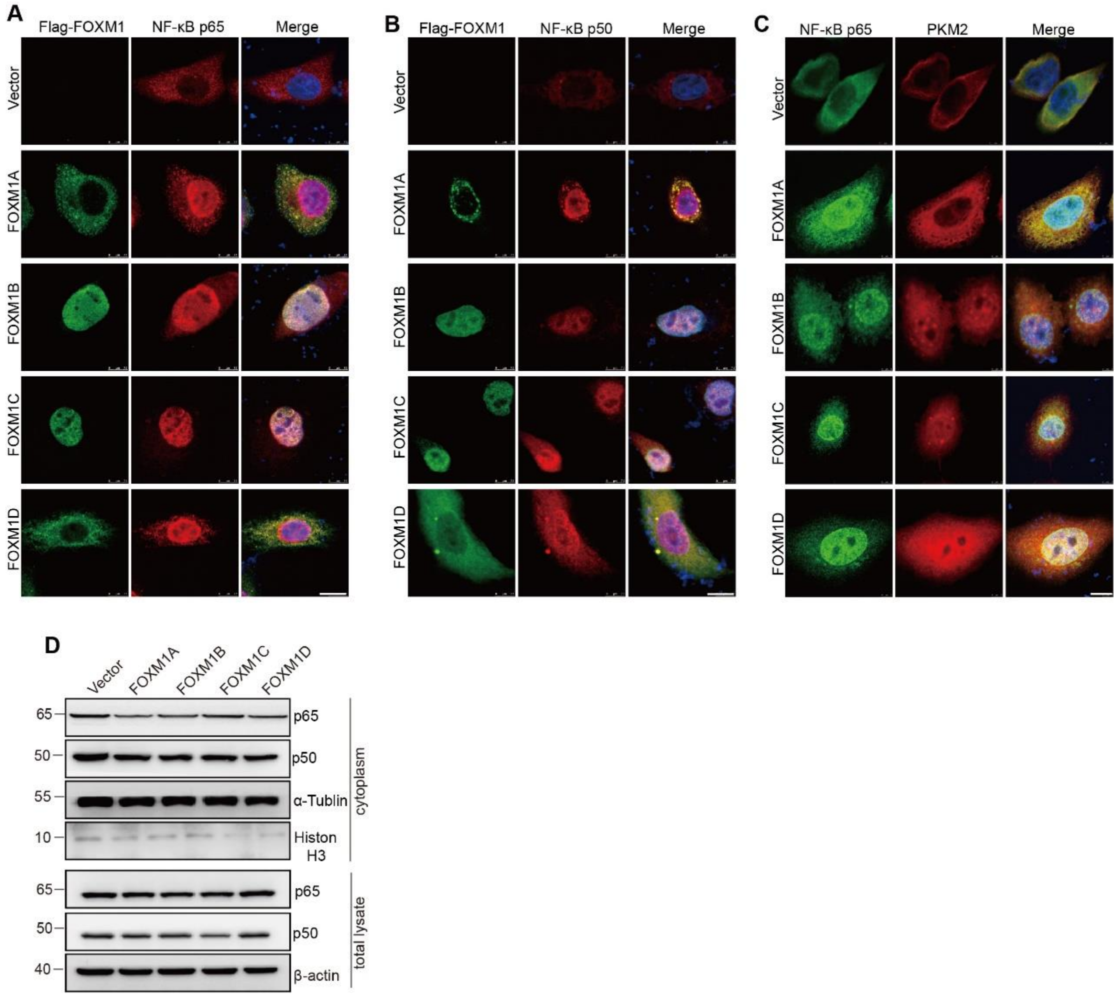

Fig. S5 Identification of the interaction between FOXM1A/B/C/D and NF-κB.

A-B The subcellular co-localization of Flag-FOXM1A/B/C/D and NF-κB p65 (A) or p50(B) subunit, the nuclear translocation of NF-κB increased upon the ectopic expression of FOXM1A/B/C/D as determined by ICC assay in HeLa cells. Scale bar, 10 μm. **C** The subcellular co-localization of NF-κB p65 and PKM2 in HeLa-overexpression FOXM1A/B/C/D and vector. Scale bar, 10 μm. **D** The subcellular detection of NF-κB by immunoblotting in HeLa cells with the ectopic expression of FOXM1A/B/C/D.

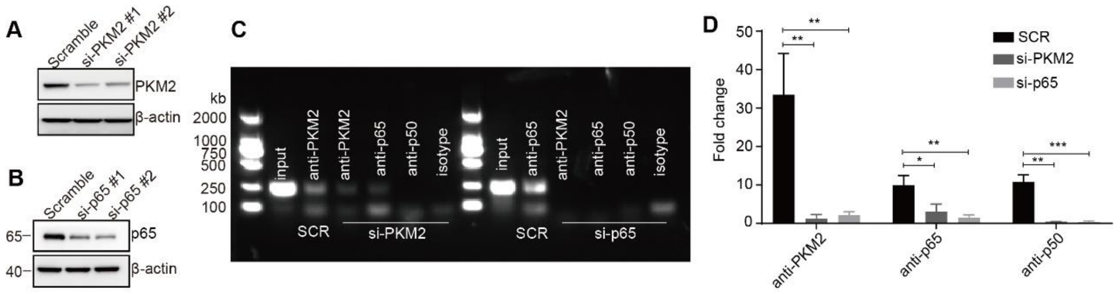

Fig. S6 FOXM1D Upregulates VEGFA Expression Mediated by PKM2 and NF-κB. **A-B** Insufficiency of PKM2 and p65 via siRNAs in HeLa-FOXM1D cells. **C** Samples with si-PKM2 #1 and si-p65 #1 were amplified by the same specific primers (Supplementary Table S2) using PCR in the ChIP assay in si-PKM2 and si-p65 in HeLa-FOXM1D cells. the isotype was the negative control using IgG antibody. **D** The quantitative data of (**C**). Data represent the means ± SD, n=3, ** p<0.01, *** p<0.001.

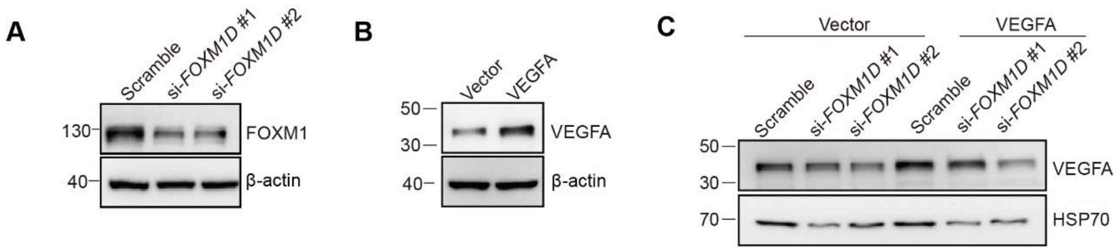

Fig. S7 FOXM1D regulates VEGFA release via exosome. **A-B** The knockdown efficiency of FOXM1D (A) and the ectopic expression efficacy of VEGFA (B) were confirmed by Western Blot in HeLa cells. **C** The exosomes were harvested from the indicated HeLa cells to detect the levels of VEGFA and HSP70 by Western blot.
