# Supplementary material for: FOXM1D potentiates PKM2‐mediated tumor glycolysis and angiogenesis
Source: Mol Oncol. 2021 Apr 2;15(5):1466–85. doi: 10.1002/1878-0261.12879 (PMC8096781; doi:10.1002/1878-0261.12879)
Supplement: Supplementary file 2 — Table S1. Information on antibodies used in the study. [file MOL2-15-1466-s002.docx]

**Table S1 Information on antibodies used in the study.**

| **Antibody** | **Source and Identifier** |
| --- | --- |
| HA | abcam; #ab18181 |
| Flag | Cell Signaling Technology;#2368 |
| GST | Cell Signaling Technology; #2625S |
| PKM2 | Cell Signaling Technology;#4053 |
| FOXM1 | santa cruze;sc-271746 |
| Histone H3 | abcam;ab133457 |
| α-tublin | proteintech;HRP-66031 |
| β-actin | santa cruze;sc-47778 |
| Phospho-PKM2 (Tyr105) | Cell Signaling Technology; #3827 |
| Phospho-PKM2 (s37) | Signalway Antibody ; #11456 |
| p50 | Cell Signaling Technology; #13586 |
| p65 | Cell Signaling Technology; #8242 |
| VEGFA | abcam;#EP1176Y |
| HSP70 | SBI;EXOAB-HSP70A-1 |
| importin 4 | abcam;#ab181037 |
| Lamin B | santa cruze;sc-377000 |
| PKM1 | Cell Signaling Technology;#7067 |
| VPS11 | abcam;#170869 |
| FBP1 | abcam;ab109732 |
| CD63 | proteintech;25682-1-AP |
