# Supplementary material for: FOXM1D potentiates PKM2‐mediated tumor glycolysis and angiogenesis
Source: Mol Oncol. 2021 Apr 2;15(5):1466–85. doi: 10.1002/1878-0261.12879 (PMC8096781; doi:10.1002/1878-0261.12879)
Supplement: Supplementary file 3 — Table S2. Information on primers and siRNA sequence used in the study. [file MOL2-15-1466-s003.doc]

**Table S2 Information on primers and siRNA sequence used in the study.**

| **Primer** |  | **Sequence** |
| --- | --- | --- |
| FOXM1A | F primer: 5'-GTCTCCACAATTGCCCGAG-3' | |
|  | R primer:5'-CCAAAATCTCGCAGATCGC-3' | |
| FOXM1B | F primer: 5'-GGTGTTTAAGCAGCAGAAAC-3' | |
|  | R primer:5'-GCAGCACCTTGGGGGCAATGC-3' | |
| FOXM1C | F primer: 5'-CCACTGGACCCAGGGTCTCC-3' | |
|  | R primer:5'-GCAGCACCTTGGGGGCAATGC-3' | |
| FOXM1D | F primer: 5'-CAGGTGTTTAAGCAGCAGA-3' | |
|  | R primer:5'-GGTGATGGGTGTACCAAAAT-3' | |
| FOXM1 | F primer: 5'-TGCAGCTAGGGATGTGAATCTTC-3' | |
|  | R primer:5'-GGAGCCCAGTCCATCAGAACT-3' | |
| FOXM1D II-III | F primer: 5'-CCGGAATTCGGATGAAAACTAGCCCCCGTCG-3' | |
|  | R primer: 5'CGGGGTACCCTATAACCTGTCGCTGCTCCAGG-3' | |
| FOXM1D II-VI | F primer: 5'-CCGGAATTCGGATGAAAACTAGCCCCCGTCG-3' | |
|  | R primer: 5'-CGGGGTACCCTAGTGCGCCCAGGGGGAGTTCG-3' | |
| FOXM1D IV-VI | F primer: 5'-CCGGAATTCGGAGGTTGAGGAGCCTTCGAGA-3' | |
|  | R primer: 5'-CGGGGTACCCTAGTGCGCCCAGGGGGAGTTCG-3' | |
| FOXM1D II-VIIa | F primer: 5'-CCGGAATTCGGATGAAAACTAGCCCCCGTCG-3' | |
|  | R primer: 5'-CGGGGTACCCTATAAACAAAGAAAGATAAAAT-3' | |
| FOXM1D VIIa-VIII | F primer: 5'-CCGGAATTCGGAGGTTTTTGGGGAACAGGTG-3' | |
|  | R primer: 5'-CGGGGTACCCTACTGTAGCTCAGGAATAAACT-3' | |
| FOXM1D VIII | F primer: 5'-CCGGAATTCGGCCTCAGCTAGCAGCACCTGA-3' | |
|  | R primer: 5'-CGGGGTACCCTACTGTAGCTCAGGAATAAACT-3' | |
| PKM2(1-388) | F primer: 5'-CGCGGATCCATGTCGAAGCCCCATAGT-3' | |
|  | R primer: 5'-CCGGAATTCGGCAGCCTCTGCCTCACG-3' | |
| PKM2(44-388) | F primer: 5'-CGCGGATCCATGACTGGCATCATCT-3' | |
|  | R primer: 5'-CCGGAATTCGGCAGCCTCTGCCTCACG-3' | |
| PKM2(44-218) | F primer: 5'-CGCGGATCCATGACTGGCATCATCT-3' | |
|  | R primer: 5'-CCGGAATTCCAAGTCCACAGC-3' | |
| PKM2(219-388) | F primer: 5'-CGCGGATCCATGCCTGCTGTGTCGGAG-3' | |
|  | R primer: 5'-CCGGAATTCGGCAGCCTCTGCCTCACG-3' | |
| PKM2(389-531) | F primer: 5'-CGCGGATCCATGATCTACCACTTGCAA-3' | |
|  | R primer: 5'-CCGGAATTCCGGCACAGGAACAACACG-3' | |
| PKM2 A1 domain | F primer: 5'-CGCGGATCCATGACTGGCATCATCT-3' | |
|  | R primer: 5'-CCGGAATTCAGGTCCTTTAGTGTCT-3' | |
| PKM2 B domain | F primer: 5'-CGCGGATCCATGGAGATCCGAACT-3' | |
|  | R primer: 5'-CCGGAATTCCAAGTCCACAGCAG-3' | |
| PKM2 | F primer: 5'-TTGCAATTATTTGAGGAACTCCGC-3' | |
|  | R primer: 5'-TGGTGAGGACGATTATGGCCCCAC-3' | |
| VEGFA | F primer: 5'-AGGGCAGAATCATCACGAAGT-3' | |
|  | R primer: 5'-AGGGTCTCGATTGGATGGCA-3' | |
| β-actin | F primer: 5'-ACCGAGCGCGGCTACAG-3' | |
|  | R primer: 5'-CTTAATGTCACGCACGATTTCC-3' | |
| VPS11 | F primer: 5'-CGGCGCTTCGTTTTCTTCG-3' | |
|  | R primer: 5'-CCCGTAGTTTGTAGGCTTGGAA-3' | |
| IPO4 | F primer: 5'-CTCTGCGACCAGGTAGACGA-3' | |
|  | R primer: 5'-CCCATCAGACCCGATAAGGC-3' | |
| VEGFA promoter binding site | F primer: 5'-TCCACCCGCCCCCACCAGCCCCC-3' | |
|  | R primer:5'-GGAGAAGAATTTGGCACCAAGT-3' | |
| Importin4-siRNA #1 sense | 5’-GCAUUUCGCUGUACAAGUUTT-3’ | |
| Importin4-siRNA #1 anti-sense | 5’-AACUUGUACAGCGAAAUGCTT-3’ | |
| Importin4-siRNA #2 sense | 5’-AGUCAGAGGUGCCGGUCAUTT-3’ | |
| Importin4-siRNA #2 anti-sense | 5’-AUGACCGGCACCUCUGACATT-3’ | |
| VPS11-siRNA #1 sense | 5’-GCCAACUGUUGUAUCUUGUTT-3’ | |
| VPS11-siRNA #1 anti-sense | 5’-ACAAGAUACAACAGUUGGCTT-3’ | |
| VPS11-siRNA #2 sense | 5’-GUCCAGCAAUAUAUCCGAATT-3’ | |
| VPS11-siRNA #2 anti-sense | 5’-UUCGGAUAUAUUGCUGGACTT-3’ | |
| PKM2-siRNA #1 sense | 5’-UGCCAUCUACCACUUGCAATT-3’ | |
| PKM2-siRNA #1 anti-sense | 5’-UUGCAAGUGGUAGAUGGCATT-3’ | |
| PKM2-siRNA #2 sense | 5’-UCCUUCAAGUGCUGCAGUGTT-3’ | |
| PKM2-siRNA #2 anti-sense | 5’-CACUGCAGCACUUGAAGGATT-3’ | |
| p65-siRNA #1 sense | 5’-GCACCAUCAACUAUGAUGATT-3’ | |
| p65-siRNA #1 anti-sense | 5’-UCAUCAUAGUUGAUGGUGCTT-3’ | |
| p65-siRNA #2 sense | 5’-GGAGUACCCUGAGGCUAUATT-3’ | |
| p65-siRNA #2 anti-sense | 5’-UAUAGCCUCAGGGUACUCCTT-3’ | |
| FOXM1D-siRNA #1 sense | 5’-CCCAUCACCAGCUUGUUUATT-3’ | |
| FOXM1D-siRNA #1 anti-sense | 5’-UAAACAAGCUGGUGAUGGGTT-3’ | |
| FOXM1D-siRNA #2 sense | 5’-CAGGUGGUGUUUGGUUACATT-3’ | |
| FOXM1D-siRNA #2 anti-sense | 5’-UGUAACCAAACACCACCUGTT-3’ | |
| Scrambled Control sense | 5’-UUCUCCGAACGUGUCACGUTT-3’ | |
| Scrambled Control anti-sense | 5’-ACGUGACACGUUCGGAGAATT-3’ | |
